# Human Campylobacteriosis in Luxembourg, 2010–2013: A Case-Control Study Combined with Multilocus Sequence Typing for Source Attribution and Risk Factor Analysis

**DOI:** 10.1038/srep20939

**Published:** 2016-02-10

**Authors:** Joël Mossong, Lapo Mughini-Gras, Christian Penny, Anthony Devaux, Christophe Olinger, Serge Losch, Henry-Michel Cauchie, Wilfrid van Pelt, Catherine Ragimbeau

**Affiliations:** 1 National Health Laboratory (LNS), Surveillance and Epidemiology of Infectious Diseases, 1 rue Louis Rech, Dudelange L-3555, Luxembourg; 2 National Institute for Public Health and the Environment (RIVM), Centre for Infectious Disease Control (CIb). PO Box 1 - 3720 BA Bilthoven, The Netherlands; 3 Utrecht University, Faculty of Veterinary Medicine, Department of Infectious Diseases and Immunology, Yalelaan 1, De Uithof - 3584 CL Utrecht, The Netherlands; 4 Luxembourg Institute of Science and Technology, Environmental Research and Innovation, 41 rue du Brill, L-4422 Belvaux, Luxembourg; 5 Veterinary Services Administration, Laboratory of Veterinary Medicine, 54 av. Gaston Diderich, L-1420 Luxembourg

## Abstract

Campylobacteriosis has increased markedly in Luxembourg during recent years. We sought to determine which *Campylobacter* genotypes infect humans, where they may originate from, and how they may infect humans. Multilocus sequence typing was performed on 1153 *Campylobacter jejuni* and 136 *C. coli* human strains to be attributed to three putative animal reservoirs (poultry, ruminants, pigs) and to environmental water using the asymmetric island model. A nationwide case-control study (2010–2013) for domestic campylobacteriosis was also conducted, including 367 *C. jejuni* and 48 *C. coli* cases, and 624 controls. Risk factors were investigated by *Campylobacter* species, and for strains attributed to different sources using a combined case-control and source attribution analysis. 282 sequence types (STs) were identified: ST-21, ST-48, ST-572, ST-50 and ST-257 were prevailing. Most cases were attributed to poultry (61.2%) and ruminants (33.3%). Consuming chicken outside the home was the dominant risk factor for both *Campylobacter* species. Newly identified risk factors included contact with garden soil for either species, and consuming beef specifically for *C. coli*. Poultry-associated campylobacteriosis was linked to poultry consumption in wintertime, and ruminant-associated campylobacteriosis to tap-water provider type. Besides confirming chicken as campylobacteriosis primary source, additional evidence was found for other reservoirs and transmission routes.

Campylobacteriosis is the leading cause of human bacterial gastroenteritis and the most reported zoonosis in the European Union (EU)^1^,^2^. Reported cases are only the ‘tip of the iceberg’ of the actual magnitude of campylobacteriosis in the population. As an example, 198,252 campylobacteriosis cases were reported in the EU in 2009, but accounting for underreporting led to an estimated 9.2 million cases^1^.

In Luxembourg, campylobacteriosis has increased dramatically during recent years, reaching an incidence of 138 cases/100,000 population in 2011, a national record and the second highest in the EU^2^. While the high incidence in 2011 was likely to be influenced by higher rates of stool testing in the wake of the EHEC O104:H4 outbreak in Germany, which also affected some Luxembourgish residents^3^, Luxembourg’s food safety authorities declared *Campylobacter* as a national priority^4^. Because approximately 90% of cases in Luxembourg are caused by *Campylobacter jejuni* and the rest is primarily caused by *C. coli*, this article focuses on *C. jejuni* and *C. coli*, and hereafter *Campylobacter* refers to these two species only.

Virtually all warm-blooded animals, and particularly avian species, are amplifying hosts for *Campylobacter*, whose presence is usually asymptomatic in animals. *Campylobacter* transmission to humans from the animal reservoirs may occur through multiple routes, including contaminated food (especially poultry meat) and water, the environment, and animal contact^5^,^6^,^7^. Although person-to-person transmission is deemed uncommon^8^, exotic *Campylobacter* strains may spread into the domestic population via returning travellers^9^.

While evidence for host-adapted *Campylobacter* strains exists^10^,^11^, predicting the host from genotype is challenging; *Campylobacter* populations display a weak clonal structure^4^,^12^ and certain sub-populations are generalists^13^. Multilocus sequence typing (MLST) has proved useful in tracing the sources of campylobacteriosis up to the animal reservoirs. Several MLST-based source attribution analyses have identified chicken as the main reservoir for *Campylobacter*, accounting for 50–80% of human cases, followed by cattle (20–30%)^5^,^10^,^14^,^15^,^16^,^17^,^18^,^19^,^20^. Likewise, case-control studies have consistently shown consumption of chicken to be the predominant risk factor for human campylobacteriosis^7^,^21^,^22^. However, contrasting findings have also been reported, with the regular consumption of poultry meat at home being identified as a protective factor^23^. This possibly reflects the effect of acquired immunity against clinical disease, but not necessarily colonization, due to repeated exposures to (and mainly asymptomatic infection with) *Campylobacter* within the household^24^.

While MLST-based source attribution analyses suggest that up to 80% of human campylobacteriosis cases originate from the chicken reservoir, only 42% of chicken-associated human *Campylobacter* strains may be attributed to chicken consumption *per se*^5^. This points to alternative transmission routes for these strains, such as environment-mediated spread^25^ and cross-contamination^21^. Given the complex nature of *Campylobacter* epidemiology, performing separate analyses for source attribution and risk factors is unlikely to provide exhaustive insights into where *Campylobacter* originates from. Thus, it has been proposed that source attribution combined with case-control data can bridge the gap between attributing human cases at the start of the transmission chain (i.e. reservoir level) and at the point of exposure (i.e. risk factors)^5^.

Focussing on a high-incidence country like Luxembourg, the aim of this study was to determine what *Campylobacter* genotypes infect humans, where amongst the main putative reservoirs they are likely to originate from, and how they may infect humans. This was achieved via: 1) extensive genotyping of *Campylobacter* strains from humans and a range of putative animal reservoirs (poultry, ruminants, pigs) and environmental water; 2) MLST-based source attribution of human *Campylobacter* strains to the aforesaid animal and environmental sources; 3) identification of potential risk factors for human campylobacteriosis (case-control study); and 4) combined case-control and source attribution analysis to identify risk factors for human campylobacteriosis caused by strains attributable to the different sources.

## Methods

### Human data

A nationwide case-control study of risk factors for human campylobacteriosis was conducted in the Grand Duchy of Luxembourg. In total, 1153 human cases of *C. jejuni* infection (89.4%) and 136 of *C. coli* (10.6%) were identified by the National Health Laboratory of Luxembourg (LNS) during December 2010–May 2013. The LNS serves as the national reference laboratory for enteropathogens and receives all *Campylobacter* isolates from private and hospital laboratories in Luxembourg. *Campylobacter* isolates were cultured and characterized at the species level using molecular methods^4^. MLST was performed according to the protocol for amplification and sequencing of the seven housekeeping genes of Dingle *et al.*^26^, with slight modifications^4^. Automated data analysis and library matching were performed with SeqScape^®^ v2.5 (ABI, Life Technologies, Belgium). For simplicity, we refer to the sequence type (ST) of an isolate as its genotype. This study used the *Campylobacter* PubMLST website (http://pubmlst.org/campylobacter/) of Oxford University^27^.

Non-diseased controls were recruited by a commercial survey company from 13,200 residents of a representative web-panel using quota sampling to match the age group (0–4, 5–14, 15–24, 25–44, 45–64, ≥65 years) and distribution of cases by month. Cases were asked to complete a paper-and-pencil questionnaire (appended as Supplementary Information S1) by mail collecting information on demographics, contact with animals, leisure activities, living environment, and food consumption. Exposure information for controls was collected using computer-assisted web interview and a small financial incentive was offered. Questions covered the five days prior to symptoms (cases) or interview (controls). Parents completed the questionnaire on behalf of their children. Missing values were handled using multiple imputation as described previously^7^,^28^. In total, 548 cases and 764 controls completed the questionnaire and were therefore included in the case-control study; 133 cases and 140 controls that had travelled abroad during the recall period were discarded, resulting in 415 domestic cases (367 *C. jejuni* and 48 *C. coli*) and 624 controls enrolled. All human data are available as Supplementary Information S2.

Differences in the occurrence of the five most common STs and clonal complexes (CCs) were examined for the variables age group, gender, urbanization degree of the commune of residence (urban >1000, intermediate 200–1000, rural <200 inhabitants/km^2^), and season (summer: June–August; autumn: September–November; winter: December–February; spring: March–May) using Pearson’s *χ*^2^ test. The place of residence of participants was used to infer their type of tap water provider. In Luxembourg, tap water is provided to households by the commune, Luxembourg’s basic administrative division. Some communes use only their own water wells (hereafter referred to as ‘local’ provision), some receive their tap water from regionally organised water syndicates (‘regional’ provision), and some use both provisions (http://www.eau.public.lu/eau_potable/production_distribution_responsabilites/syndicats_eau_potable.jpg). Deep groundwater aquifers (the “Grès du Luxembourg”) and the “Upper-Sûre reservoir” represent the major sources of drinking water in Luxembourg. Although most drinking water undergoes treatment by sand filtration and chlorination before distribution, some groundwater wells are likely to be used without any purification.

### Source data

A dataset was compiled including 498 *C. jejuni* and 340 *C. coli* isolates from food-producing animals (*n* = 509) and from environmental water (*n* = 329) typed using MLST as per the human isolates. Animal isolates were obtained from poultry (*n* = 338), ruminants (*n* = 109), and swine (*n* = 62). Most of these isolates were collected in Luxembourg (*n* = 799), and only few swine isolates were collected in the geographically close countries the Netherlands (*n* = 17), France (*n* = 16), and Belgium (*n* = 6). Animal isolates originated from either faeces or meat and were collected during 2003–2013 as part of different research and surveillance activities on farm, slaughterhouse, and retail (Table 1). The environmental water isolates were sourced from multiple sites in Luxembourg, including rivers, ponds, recreational waters, and wastewater treatment plant outlets. Environmental water was treated as a source in the attribution analysis (see below). Although environmental water cannot be considered as an amplifying host for campylobacters, it represents a ‘sink’ collecting strains from a variety of different hosts, including those commonly found in animals and humans^14^,^16^,^17^. Therefore, similar to previous studies^5^,^14^,^16^,^17^, environmental water was considered here as a proxy for other unidentified (animal) reservoirs, including wildlife. Part of the source isolates employed here have already been used in previous studies conducted in Luxembourg^4^,^29^ and the Netherlands^5^,^9^,^17^. Source data are available in the *Campylobacter* PubMLST website.

### Source attribution analysis

Attribution of the 1289 MLST-typed human campylobacteriosis cases to poultry, ruminants, swine, and environmental water was performed using the asymmetric island model (AIM), which is presented in detail elsewhere^15^. Briefly, the AIM uses a Bayesian approach for attributing human *Campylobacter* strains to different sources based on their MLST profiles, accounting for genetic relatedness amongst STs. The AIM represents the *Campylobacter* population as separate islands, one per each source. The population is homogeneously mixing within each island and strains can migrate between islands, determining different levels of genetic differentiation. The AIM assumes that the observed genotypes arise from mutation (where an allele at a locus is novel), recombination (where an allele at a locus has been observed before in another allelic profile), and migration (where an allelic profile has been observed elsewhere). After estimating the mutation and recombination rates within each source, and the migration rates between sources and from each source to humans, the AIM uses these rates to estimate the relative contribution of each source to human cases. Attributions rely on the calculation of sampling probabilities, i.e. the likelihood that a given human genotype is sampled from a given source. One of the major advantages of the AIM is that it can account for novel (combination of) alleles in strains from humans unobserved in source populations^15^. For every human ST, the AIM estimated a relative posterior probability (*Pr*) to come from poultry (*Pr*_P_), ruminants (*Pr*_R_), swine (*Pr*_S_), and environmental water (*Pr*_W_).

### Risk factor analysis

We conducted a risk factor analysis for human campylobacteriosis as previously performed^5^,^6^,^7^,^9^, including the 415 domestic cases and the 624 non-travelling controls. *C. jejuni* and *C. coli* cases were at first analysed together to identify risk factors for campylobacteriosis as a whole, and then separately to identify species-specific risk factors, as compared to the controls. To identify factors uniquely associated with either *Campylobacter* species, a case-case comparison was also performed using the *C. jejuni* cases as ‘controls’^7^,^30^.

For preliminary statistical testing, 48 putative risk factors (Supplementary Information S2) were tested for association with the outcome using unconditional logistic regression, with the variables age group, gender, urbanization degree, and season always included as covariates to control for confounding. Variables showing a p ≤ 0.10 were selected for inclusion in a multivariable logistic regression model built in backward stepwise fashion. Variables were dropped one by one if they showed a p ≥ 0.05 and their exclusion from the model did not influence the association of the other covariates. Biologically plausible interactions were also tested, and the model was expanded to include significant interaction terms. Selection between collinear variables was made based on improved model fit as revealed by the Akaike information criterion (AIC).

Subsequently, risk factors for human campylobacteriosis caused by *Campylobacter* STs putatively originating from poultry, ruminants, swine, and environmental water were studied by building logistic regression models including separate subsets of cases (either *Campylobacter* species combined) attributable to each of the sources based on their estimated *Pr* values. The assignment of cases to sources was performed like in previous studies^5^,^31^,^32^. A cut-off point of the *Pr* distribution for each source was determined to provide a balance between the number of cases assignable to each source and the confidence as to their correct assignment derived by the highest possible *Pr* value. The final cut-off points represented the best compromise between the increasing *Pr* for a given source (i.e. increase in source specificity) and the decreasing number of cases included in the models (i.e. decrease in statistical power and failure of the model to converge)^5^. A separate model for each group of source-assigned STs was built to identify risk factors for *Campylobacter* infection. For cases of probable poultry and ruminant origin, a *Pr* cut-off point of 0.80 was determined, resulting in the selection of 161 cases with a mean *Pr*_P_ of 0.93 (range 0.83–0.99) – hereafter referred to as poultry-assigned cases – and of 68 cases with a mean *Pr*_R_ of 0.84 (range 0.81–0.94), i.e. ruminant-assigned cases. For cases of probable swine origin, the construction of any regression model was not possible since there were only three cases with *Pr*_S_ ≥ 0.25. Moving the cut-off point to a *Pr*_S_ < 0.25 would have resulted in the inclusion of many cases nearly equally, or even mainly, attributed to the other sources, making the risk factor analysis for swine-assigned campylobacteriosis unclear and uninformative. Therefore, the swine source was not further considered here. For campylobacteriosis of probable environmental water origin, only 21 cases had a *Pr*_W_ ≥ 0.25; analyses were thus performed using these 21 cases, having a mean *Pr*_W_ of 0.38 (range 0.28–0.94). Given the limited number of environmental water-assigned cases, the above *a priori* confounders were included in the model only if they influenced the variable of interest by ≥10%. The complete list of STs and respective cases assigned to each of the sources included in the case-control study is appended as Supplementary Information S3.

Logistic regression models for poultry- and ruminant-assigned campylobacteriosis were built the same way as described above, using the 624 controls as comparison group. Finally, to identify factors uniquely associated with campylobacteriosis from poultry or ruminants, a case-case analysis was performed comparing exposures of poultry- and ruminant-assigned cases with one another. For simplicity, while all available risk factors were tested for all the outcome variables considered here, only the results of the final multivariable regression models based on the aforementioned stepwise procedure were then presented. All final models showed an overall statistical significance (likelihood ratio *χ*^2^ test, p < 0.05) and goodness-of-fit (Hosmer-Lemeshow test, p > 0.05). Statistical analyses were performed using STATA 13 (StataCorp LP, College Station, USA).

### Ethics statement

This study was approved by the National Research Ethics Committee (CNER) of Luxembourg (www.cner.lu) and was conducted in accordance with the principles of the Declaration of Helsinki. All methods were carried out in accordance with approved guidelines and regulations. Informed consent was obtained from all subjects. No subject-identifiable data were generated.

## Results

### Clinical findings and travel information

Most campylobacteriosis cases reported to have had diarrhoea (97.2%), abdominal cramps (91.1%), fever (61.3%), headache (59.5%), malaise (53.3%), blood in the stool (45.8%), and vomiting (24.8%). *C. coli* cases were more likely to report vomiting than *C. jejuni* cases (37.0% *vs*. 23.3%, *χ*^2^ test, p = 0.027). Median duration of illness was 8 days for both *C. jejuni* and *C. coli*. Of the *C. jejuni* and *C. coli* cases, 74 (15.6%) and 7 (11.7%) were hospitalized for a median duration of 4 and 1 days, respectively (Mann-Whitney U test, p = 0.034). Travelling abroad was reported by 120 *C. jejuni* (25.1%) and 13 *C. coli* cases (21.7%), and this was significantly associated with campylobacteriosis (odds ratio [OR] 1.46, 95% confidence interval [95%CI] 1.11–1.93). Most travellers belonged to the 25-44 (28%) and 45-64 (23%) year-old age groups, whereas the age groups with the smallest proportions of travellers were those at the edges: ≥65 (7%) and ≤4 (8%) years. For 27.3% of the travel cases *vs*. 3.6% of the travel controls, the primary travel destination was a non-EU country (OR 10.13, 95%CI 3.74-34.00).

### *Campylobacter* genotypes in humans

**Figure 1:**
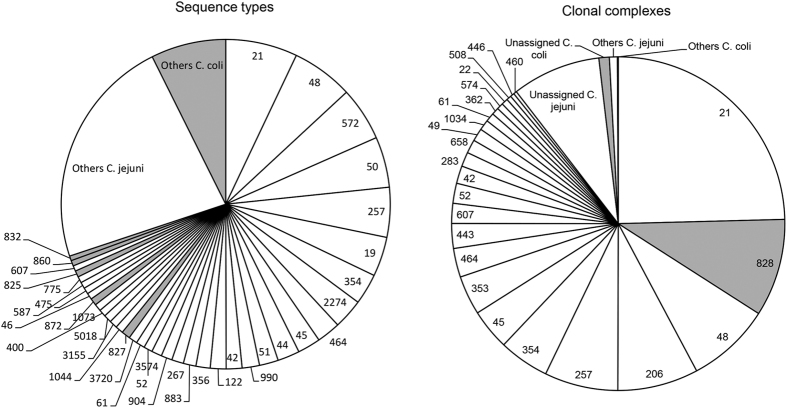
Human *Campylobacter* sequence types and clonal complexes. The category ‘others’ includes sequence types with ≤5 isolates and clonal complexes with ≤3 isolates. White segments refer to *C. jejuni*, grey segments to *C. coli*.

The 1289 MLST-typed human *Campylobacter* strains were assigned to 282 STs belonging to 34 CCs. Eighty-four STs could not be assigned to a previously identified CC. The five most frequent STs, ST-21, ST-48, ST-572, ST-50 and ST-257, accounted for 28.3% of human isolates, while the five most frequent CCs, CC-21, CC-828, CC-48, CC-206 and CC-257, accounted for 56.3% of human isolates (Fig. 1). While *C. jejuni* ST and CC frequencies followed the above ranking, the top five *C. coli* STs were ST-827, ST-872, ST-825, ST-860 and ST-832, all belonging to CC-828. Significant seasonal effects were found for ST-48 (*χ*^2^ test, p = 0.035) and its CC, CC-48 (p = 0.030), which showed the lowest frequencies in autumn (6.9% and 6.3%, respectively) and peaked in summer (48.3% and 46.9%, respectively). No significant effects of age, gender, and urbanization were found for the other STs and CCs.

### Attribution of human campylobacteriosis

**Figure 2:**
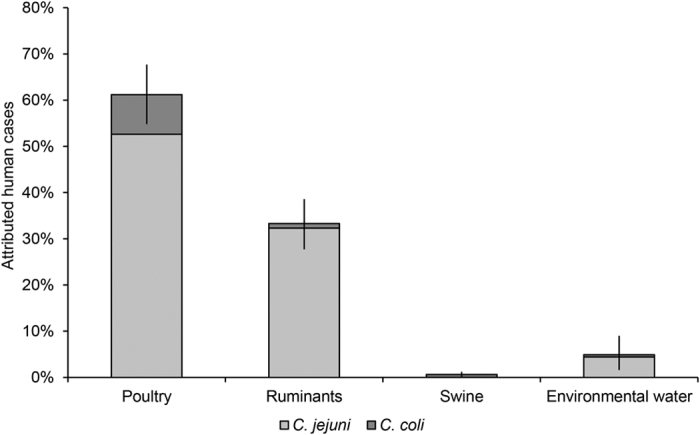
Percentage of human *C. jejuni* (*n* = 1152) and *C. coli* (*n* = 136) cases attributed to poultry, ruminants, swine, and environmental water. Error bars represent 95% confidence intervals.

**Figure 3:**
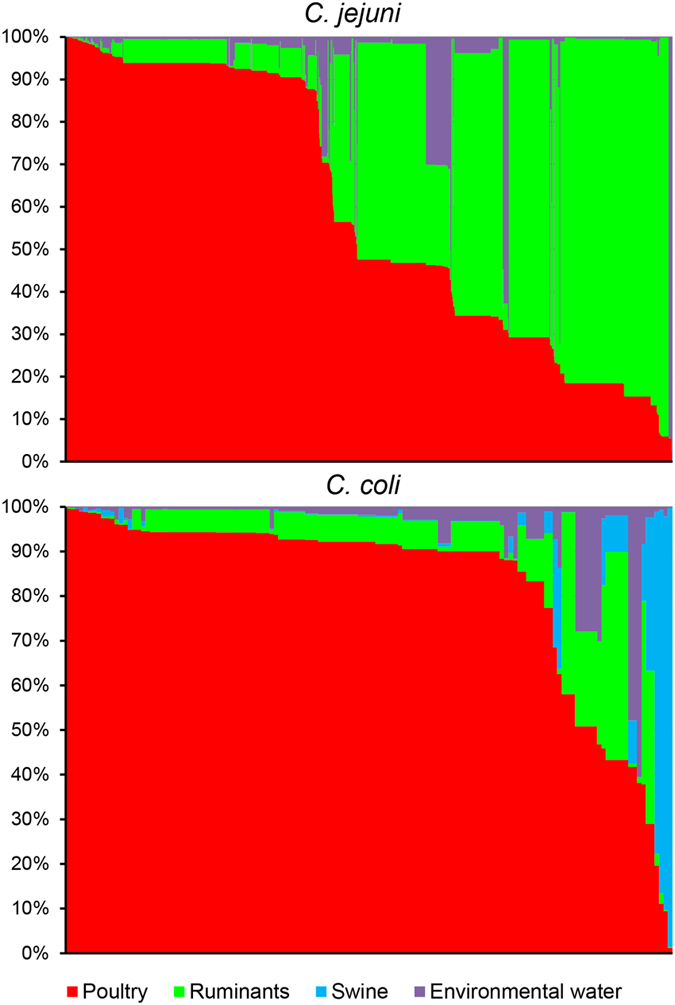
Estimated source probability matrix plot for *C. jejuni* and *C. coli*. Each human case is a vertical column with level of shading according to the probability that it originated from each of the sources. To aid visualization, cases are ordered horizontally according to the chicken source probability.

The AIM attributed the majority of human cases to poultry (61.2%, 95%CI: 54.8–67.7%), followed by ruminants (33.3%, 95%CI: 27.7–38.6%), environmental water (4.9%, 95%CI: 1.6–9.0%), and swine (0.6%, 95%CI: 0.1–1.2%) (Fig. 2). The 1153 *C. jejuni* cases were attributed as follows: poultry, 58.8% (95%CI: 54.5–63.5%); ruminants, 36.3% (95%CI: 32.9–39.9); environmental water, 4.9% (95%CI: 3.7–6.3%); and swine, 0.02% (95%CI: 0.00–0.32%). Attributions of the 136 *C. coli* cases were: poultry, 82.4% (95%CI: 68.0–99.1%); ruminants, 8.8% (95%CI: 4.5–15.3%); environmental water, 4.5% (95%CI: 1.6–9.5%); and swine 4.4% (95%CI: 1.6–9.5%) (Figs 2 and 3). Significant differences between *C. jejuni* and *C. coli* were found for *Pr*_P_ (Mann-Whitney U test, p < 0.001), *Pr*_R_ (p < 0.001), and *Pr*_S_ (p < 0.001).

**Figure 4:**
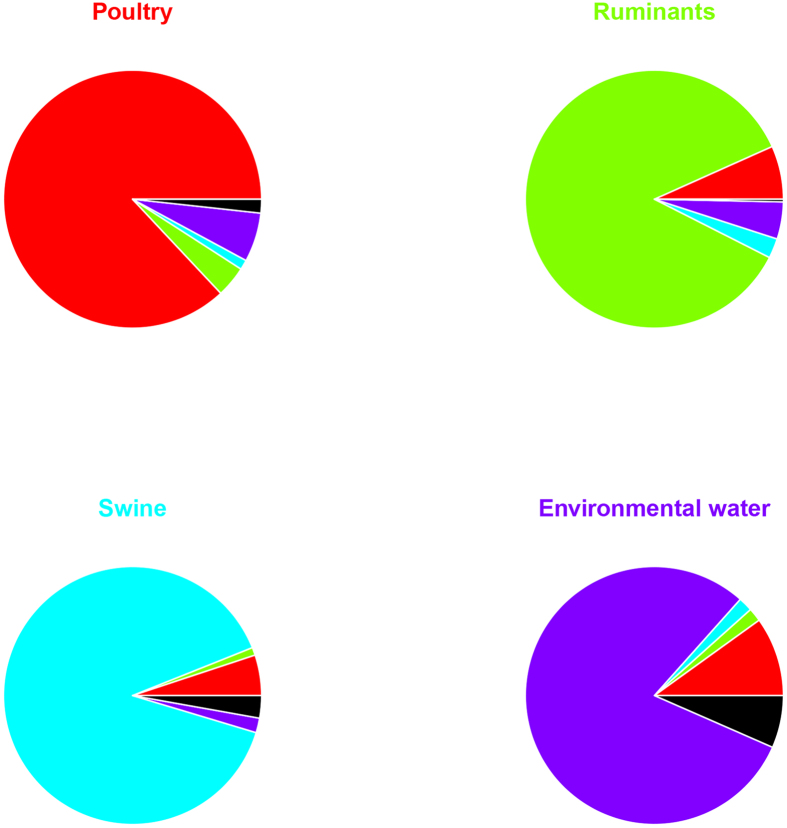
Migration and mutation rates of the different sources. The pie charts show the probability that a newly sampled allele is a novel mutant (black segment) or identical to one already observed in the same or another source (segment coloured according to the colour of the source name).

Figure 4 shows the pie charts representing the probability that a newly sampled allele at a source is a mutant (black segment) or a migrant from the same or another source (coloured segment). Estimated recombination rates were 5.4% (poultry), 2.6% (ruminants), 30.0% (swine), and 7.7% (environmental water). Posterior assignment probabilities of STs to sources are appended as Supplementary Information S3.

### Risk factors for human campylobacteriosis

The significant risk factors for human campylobacteriosis as a whole (*C. jejuni* and *C. coli* combined) are reported in Table 2. Consuming chicken outside the home (OR 2.10), and both at home and outside (OR 4.77), but not at home only, were associated with campylobacteriosis. Consuming poultry other than chicken outside the home was also associated with campylobacteriosis (OR 1.98). Campylobacteriosis was also more likely to occur in summer than winter (OR 2.00), and in those who had had contact with garden soil (OR 2.94).

Consuming organic fruit, contact with fresh produce, performing outdoor sport/recreational activities, and contact with dogs outside the home were protective factors (OR 0.64, 0.37, 0.54, and 0.51 respectively) (Table 2).

### Risk factors for human *C. jejuni* and *C. coli* infections

The significant risk factors for *C. jejuni* infection (Table 2) resembled those for campylobacteriosis in general. These were: consuming chicken outside the home (OR 2.05) and both at home and outside (OR 4.52), consuming poultry other than chicken outside the home (OR 2.01), contact with garden soil (3.03), and summertime (OR 1.92). Protective factors for *C. jejuni* infection were consuming organic fruit (OR 0.64), contact with fresh produce (0.38), performing outdoor sport/recreational activities (0.52), and contact with dogs outside the home (0.54).

For *C. coli* infection (Table 2), risk factors were consuming chicken outside the home (OR 3.02) and both at home and outside (OR 6.33), consuming beef at home (OR 2.89) and both at home and outside (OR 3.54), and consuming hamburger outside the home (OR 2.74). Female gender was associated with *C. coli* infection (OR 2.36), and so were the age groups 25–44 years (OR 7.64), 45–64 years (OR 6.27), and ≥65 years (OR 8.03) *vs*. the ≤4-year-olds. The only protective factor for *C. coli* infection was contact with fresh produce (OR 0.20).

In the case-case comparison (Table 2), the only factor associated with *C. coli* infection besides female gender (OR 2.05) was consuming beef both at home and outside (OR 5.04).

### Risk factors for poultry-assigned human campylobacteriosis

Significant risk factors for poultry-assigned campylobacteriosis were eating chicken in winter (OR 3.13), eating poultry other than chicken in winter (OR 3.28) and in autumn (OR 2.70), and contact with garden soil (OR 3.18) (Table 3). Protective factors for poultry-assigned campylobacteriosis were contact with fresh produce (OR 0.37), drinking tap water (OR 0.50), and performing outdoor sport/recreational activities (OR 0.63). The risk for poultry-assigned campylobacteriosis was higher in autumn (OR 2.96), spring (OR 3.78), and summer (OR 5.81) than in winter. Such risk was also higher in the age groups 15–24 years (OR 2.52) and 25–44 years (OR 2.30) *vs*. the ≤4-year-olds (Table 3).

Comparing exposures of poultry-assigned cases *vs*. ruminant-assigned cases highlighted one risk factor and one protective factor: eating chicken in wintertime (OR 7.28, 95%CI 1.58–33.56) and drinking tap water (OR 0.40, 95%CI 0.20–0.84).

### Risk factors for ruminant-assigned human campylobacteriosis

Contact with garden soil and having both a local and a regional tap water provider in the household (as compared to having a local provider only) were the only risk factors for ruminant-assigned campylobacteriosis (OR 2.98 and 3.80, respectively) (Table 3). Protective factors were contact with fresh produce (OR 0.45), eating grilled sausages (OR 0.49), and performing outdoor sport/recreational activities (OR 0.49). The risk for ruminant-assigned campylobacteriosis was higher in summer than winter (OR 2.94).

### Risk factors for environmental water-assigned human campylobacteriosis

The only factor associated with campylobacteriosis of probable environmental water origin was contact with garden soil (OR 3.27) (Table 3).

## Discussion

This study was performed to determine the *Campylobacter* genotypes causing human disease, their likely sources, and the associated risk exposures in Luxembourg, a country with a high campylobacteriosis incidence. A large number of cases (and controls), as well as non-human *Campylobacter* isolates, was included in this study, allowing for a multitude of analyses to infer the sources of human cases and to study source-specific risk factors in addition to those by *Campylobacter* species.

Source attribution analysis confirmed that poultry is the main reservoir for both *C. jejuni* and *C. coli*, though a relatively high contribution from cattle to *C. jejuni* and from swine to *C. coli* was found, supporting previous evidence that *C. jejuni* is more prevalent than *C. coli* in cattle and that the inverse holds for swine^5^,^33^,^34^.

With 282 STs identified amongst 1289 human cases, our results indicate a considerable diversity of genotypes in Luxembourg’s *Campylobacter* population. Rare genotypes were also relatively frequent, as STs occurring ≤5 times accounted for 27% of all cases. The top five STs and CCs identified here have been reported worldwide^5^,^16^,^19^,^26^,^35^,^36^,^37^,^38^,^39^ and rank amongst the top genotypes of other EU countries^5^,^19^,^37^,^39^, suggesting that these genotypes are endemic also in Luxembourg. Although ST-48 and its CC seemed to occur more often in summer, the other predominant genotypes were evenly distributed over demographic groups, urbanization degrees and seasons.

Poultry was confirmed to be the primary source of campylobacteriosis in Luxembourg, accounting for 61% of cases, followed by ruminants (33%). This ranking is in line with other studies from developed countries using the AIM^5^,^15^,^16^,^18^,^20^,^33^. Interestingly, our fraction of cases attributed to ruminants was somewhat high, but comparable to previous research from Switzerland^33^ and Scotland^20^ where 36% and 38% of *C. jejuni* cases were attributable to ruminants, respectively. Altogether, these findings support the growing body of evidence (reviewed by Stanley and Jones^40^) indicating that the significance of non-poultry sources should not be underestimated. *Campylobacter* colonization of ruminants does not only relate to the potential contamination of meat and dairy products, but also to contamination of the environment, including water, from disposal of slaughterhouse effluents and farm slurry/manure. Contaminated irrigation water can transfer *Campylobacter* to growing leafy vegetables and herbs^41^. Ruminants are often implicated in cases of drinking water contamination with *Campylobacter* from agricultural run-off^42^. Our findings that drinking tap water was associated with increased risk for infection with ruminant-assigned *vs*. poultry-assigned STs and that the risk of infection with ruminant-assigned STs was higher in households with both a regional and a local water supplier, warrant further attention on this exposure.

While source attribution analysis quantified the relative contributions of the different sources to human cases, the risk factor analysis identified factors associated with increased or decreased risk for infection. Risk factors could be analysed in more detail for groups of STs with high probability to originate from a specific source, allowing for the identification of possible pathways by which these STs may have reached humans from their attributed sources. We found consuming chicken and other poultry to be a risk factor only when these were consumed outside the household, and for chicken the effect size was even higher when this was consumed both at home and outside, suggesting an effect of (multiple) exposures to chicken-assigned campylobacters besides domestic food handling and consumption. We hypothesized that this risk may be due to increased chance of encountering (higher doses of) *Campylobacter* strains different from those to which people are (usually) exposed in their kitchen, as repeated exposure to *Campylobacter* strains may lead to sufficient immunity to provide protection against (severe) clinical illness^24^. Whereas these differences were not so pronounced when splitting cases according to their attributed reservoirs, we found significant seasonal effects in the risk for poultry-assigned STs. The higher risk derived from consumption of chicken in winter as compared to summer was quite unexpected, as we hypothesized that this risk would be higher in summer since *Campylobacter* incidence in broilers follows the temperature pattern^43^. The increased risk posed by consuming chicken or other poultry during wintertime may therefore be due to whom (regular/irregular consumers) and to the manner this poultry is consumed in winter. It has recently been suggested that meat fondues, such as ‘fondue chinoise’, a popular dish that is traditionally consumed during the festive season in countries like Switzerland and Germany, are a major driver of the winter epidemic peak of campylobacteriosis^44^. At these meals, the individual handling/preparation of raw poultry meat directly at the point of consumption poses a high risk for *Campylobacter* transmission and cross-contamination. Since meat fondues are popular in Luxembourg, the seasonal effect observed here might well be, to some extent, the result of this culinary tradition.

Surprisingly, we found that consuming beef (both at home and outside) was a distinct risk factor for *C. coli* infection. The risk for *C. coli* infection posed by consumption of beef products, particularly offal and tripe, has been reported before^7^. There is, however, little evidence that consumption of red meat poses a risk for campylobacteriosis in general^5^, as it is rarely contaminated with *Campylobacter*, and where contamination exists, it is at low doses^45^. Moreover, we found that female gender and older age groups had an increased risk for *C. coli* infection, possibly reflecting some hitherto unknown exposures linked to typically female- and adulthood-oriented tasks and eating habits.

Contact with garden soil stood out as a novel and consistent risk factor for *Campylobacter* infection, irrespective of the attributed reservoirs. Garden soil is often obtained by aerobic composting in which the organic matter in manure and other substrates is transformed and stabilized into humus-like products. When performed properly, this process eliminates coliform bacteria^46^, parasite (oo)cysts^47^, and some viruses^48^. Although *Campylobacter* does not survive well in solid manure^48^,^49^, and compost is a very inhospitable environment, recent research has demonstrated that *Campylobacter* in (cattle) faeces can persist in compost for prolonged periods, calling into question the common belief that *Campylobacter* has poor fitness outside the host^50^. Further research into this newly identified potential at-risk exposure is therefore warranted.

Consumption of organic fruit, contact with (and presumably consumption of) fresh produce, and performing outdoor sport/recreational activities were protective factors. These are not unusual findings^5^,^7^,^21^,^22^, as it is generally believed that a diet rich in fruit and vegetables may have genuinely beneficial effects on health, and performing outdoor sport/recreational activities may be a proxy of healthy living. However, selection bias might also have occurred, as our controls might have been particularly motivated people with a generally healthier lifestyle. An advantage of having performed the case-case analysis was that selection (and recall) bias were minimised. Another protective factor was contact with dogs outside the household. This has been found in other studies^5^,^6^, and a possible explanation is that contact with dogs other than their own encourages people to undertake protective actions such as hand washing^5^,^6^.

A limitation of this study was the residual contribution to *Pr* values from sources other than those to which human cases were assigned. To address this, regression models were restricted to subsets of cases with the highest possible *Pr* for each source. However, this residual contribution, although minimized, could have masked or diluted some associations, or led to some additional associations, in the risk factor analysis. The latter option could be the case of consuming grilled sausages as a protective factor for ruminant-assigned campylobacteriosis, though sausages are more commonly made with pork than ruminant meats. We did not have information on underlying conditions and medication use, such as proton-pump inhibitors, that may have had an effect on campylobacteriosis, being therefore a major weakness of this study. For practical reasons, we could not test the controls for asymptomatic *Campylobacter* infection, which might lead to some misclassification. However, campylobacteriosis cases were identified by passive surveillance of gastroenteritis; thus, they represented the most severe infections occurring in the population. It follows, therefore, that our results mainly refer to severe campylobacteriosis. The human and source isolates were not perfectly contemporaneous, but this is unlikely to be a cause for concern. Indeed, Smid *et al.*^17^ showed that while the MLST frequencies of two sources become increasingly dissimilar over time, this is far less problematic than the potential bias introduced by using data from geographically distant regions. We therefore used all the locally sourced (albeit not always fully contemporaneous) data at our disposal to minimize bias and reach satisfactory statistical power^17^. Moreover, the source isolates were obtained during or before (but not after) the human isolates; thus, given the unidirectional transmission (from sources to humans) on which the attribution implicitly relies, attributing human cases based on what goes or went on in the sources (i.e. the current and previous STs circulating in sources), rather than the other way around, would theoretically make the consequences of any temporal bias less severe than when attributing cases forward in time. While our sampling reflected the actual animal production in Luxembourg, we supplemented the local pig isolates with some isolates from Luxembourg’s neighbouring countries, with which Luxembourg has intensive trading relationship of foodstuffs, including pork products. Therefore, the non-local pig strains were very representative of what could be found in Luxembourg’s food market. Accordingly, bias due to the use of non-local data can be minimized by using data from geographically close areas^17^. Finally, for logistic reasons, cases and controls were interviewed in a different way, i.e. paper questionnaire for cases and web-assisted interview for controls. This might have influenced the participation (as this usually tends to be lower in web-based questionnaires, although we compensated for that by offering a financial incentive to controls) as well as the recall, which was probably more accurate for cases since they had more time and opportunities to remember and possibly correcting the answers, potentially leading to the exaggeration of some of the effects seen here.

In conclusion, MLST allowed us to investigate *Campylobacter* diversity in Luxembourg, though the predominant genotypes in humans were also typical of other EU countries. Regarding where these strains came from, we provided evidence that interventions against *Campylobacter* should not only focus on poultry, but also on ruminants. This has significant implications since there has been some reluctance to accept that also non-poultry *Campylobacter* strains pose a substantial public health burden^40^. Several risk factors were assessed to ascertain how *Campylobacter* might infect humans, and some of the identified risk factors were novel (e.g. consuming beef, contact with garden soil, and tap water provider type) or presented in a somewhat more nuanced way (e.g. consuming poultry within or outside the household) than has been shown previously. Combining epidemiological and genotype-based source attribution data was helpful in enhancing risk factor characterization. This approach helped supporting and generating hypotheses, such as that of the potential role of meat fondues for poultry-associated campylobacteriosis during wintertime, as well as the increased risk for ruminant-associated campylobacteriosis linked to drinking tap water and having both a local and regional water provider. From a larger perspective, this study provided insights in the public health importance of different *Campylobacter* genotypes, sources and risk factors in a high-incidence setting, enhancing our understanding of the underlying transmission pathways.

## Additional Information

**How to cite this article**: Mossong, J. *et al.* Human Campylobacteriosis in Luxembourg, 2010-2013: A Case-Control Study Combined with Multilocus Sequence Typing for Source Attribution and Risk Factor Analysis. *Sci. Rep.* **6**, 20939; doi: 10.1038/srep20939 (2016).

## Supplementary Material

Supplementary Information

Supplementary Information

## Figures and Tables

**Table 1:** MLST typed *Campylobacter* isolates used in the source attribution analysis.

Country	Humans	Poultry^1^	Ruminants^2^	Pigs	Environmental water
Luxembourg	1289	338	109	23	329
The Netherlands	—	—	—	17	—
Belgium	—	—	—	6	—
France	—	—	—	16	—

^1^Includes broiler chickens (*n* = 254), turkeys (*n* = 49), meat ducks (*n* = 3), quails (*n* = 4), Guinea fowl (*n* = 2), and other unidentified domestic poultry (*n* = 26).

^2^Includes cattle (*n* = 101), sheep (*n* = 4), and goats (*n* = 4).

**Table 2:** Multivariable odds ratios (and 95% confidence intervals) of the significant risk factors for human *C. jejuni* and *C. coli* infections.

Variable (% imputed missing values)^1^	*C. jejuni/coli vs*. controls^2^	*C. jejuni vs.*controls^3^	*C. coli vs.*controls^4^	*C. coli vs. C. jejuni*^5^
Season				
Winter (Dec-Feb)	Reference	Reference	Reference	Reference
Spring (Mar-May)	0.99 (0.67–1.47)	0.91 (0.61–1.37)	1.99 (0.75–5.30)	1.99 (0.73–5.40)
Summer (Jun-Aug)	2.00 (1.34–2.99)	1.92 (1.27–2.91)	2.63 (0.95–7.30)	1.06 (0.39–2.90)
Autumn (Sep-Nov)	1.17 (0.78–1.76)	1.10 (0.72–1.68)	2.53 (0.91–7.01)	1.96 (0.71–5.46)
Age group				
≤4 years	Reference	Reference	Reference	Reference
5–14 years	0.85 (0.50–1.44)	0.82 (0.48–1.40)	1.19 (0.19–7.47)	1.99 (0.34–11.77)
15–24 years	1.73 (0.97–3.10)	1.66 (0.91–3.00)	4.08 (0.70–23.83)	3.80 (0.70–20.63)
25–44 years	1.47 (0.90–2.39)	1.27 (0.77–2.09)	7.64 (1.44–40.71)	4.60 (0.99–21.36)
45–64 years	1.05 (0.63–1.75)	0.91 (0.54–1.52)	6.27 (1.14–34.34)	4.06 (0.84–19.59)
≥65 years	1.21 (0.68–2.16)	1.05 (0.58–1.89)	8.03 (1.31–49.37)	3.59 (0.67–19.11)
Gender (female *vs*. male)	0.98 (0.74–1.30)	0.92 (0.69–1.23)	2.36 (1.15–4.84)	2.05 (1.05–3.98)
Urbanization degree				
Urban (>1000 people/km^2^)	Reference	Reference	Reference	Reference
Intermediate (200–1000 people/km^2^)	0.87 (0.62–1.22)	0.87 (0.61–1.23)	0.93 (0.43–2.02)	1.33 (0.62–2.83)
Rural (<200 people/km^2^)	1.07 (0.75–1.52)	1.08 (0.75–1.56)	0.91 (0.40–2.11)	0.94 (0.42–2.10)
Eating chicken (4.2%)				ns
No	Reference	Reference	Reference	
Only at home	1.15 (0.83–1.60)	1.09 (0.78–1.52)	1.59 (0.65–3.93)	
Only outside the home	2.10 (1.32–3.34)	2.05 (1.27–3.30)	3.02 (1.02–8.93)	
Both at home and outside	4.77 (2.11–10.74)	4.52 (1.94–10.52)	6.33 (1.25–32.06)	
Eating poultry other than chicken (8.6%)			ns	ns
No	Reference	Reference		
Only at home	1.36 (0.97–1.91)	1.37 (0.96–1.96)		
Only outside the home	1.98 (1.11–3.56)	2.01 (1.08–3.74)		
Both at home and outside	1.78 (0.47–6.83)	1.53 (0.38–6.21)		
Eating beef (7.1%)	ns	ns		
No			Reference	Reference
Only at home			2.89 (1.06–7.89)	2.63 (0.93–7.49)
Only outside the home			0.70 (0.10–5.03)	0.76 (0.81–4.68)
Both at home and outside			3.54 (1.00–12.63)	5.04 (1.43–17.73)
Eating hamburger (5.6%)	ns	ns		ns
No			Reference	
Only at home			1.18 (0.40–3.46)	
Only outside the home			2.74 (1.12–6.69)	
Both at home and outside			3.90 (0.30–51.22)	
Eating organic fruit (9.8%)	0.64 (0.45–0.89)	0.64 (0.45–0.92)	ns	ns
Contact with fresh produce (5.5%)	0.37 (0.25–0.54)	0.38 (0.25–0.56)	0.20 (0.09–0.48)	ns
Contact with garden soil (5.4%)	2.94 (2.12–4.09)	3.03 (2.15–4.28)	ns	ns
Outdoor sport/recreational activity (4.1%)	0.54 (0.38–0.75)	0.52 (0.37–0.73)	ns	ns
Contact with a dog (4.8%)			ns	ns
No	Reference	Reference		
Only at home	1.11 (0.80–1.53)	1.12 (0.80–1.56)		
Only outside the home	0.51 (0.34–0.77)	0.54 (0.35–0.83)		
Both at home and outside	0.78 (0.34–1.78)	0.81 (0.34–1.97)		

ns = factor not significant (p > 0.05) and/or not influencing the associations of the other covariates, thereby dropped from the multivariable model.

^1^Fraction of imputed missing values in the whole data set.

^2^Based on 415 cases (*C. jejuni* and *C. coli* combined) and 624 controls.

^3^Based on 367 *C. jejuni* cases and 624 controls.

^4^Based on 48 *C. coli* cases and 624 controls.

^5^Based on 48 *C. coli* cases used as “cases” *vs*. 367 *C. jejuni* cases used as “controls”.

**Table 3:** Multivariable odds ratios (and 95% confidence intervals) of the significant risk factors for human campylobacteriosis putatively originating from poultry, ruminants and environmental water.

Risk factor (% imputed missing values)^1^	Poultry-associated cases *vs*. controls^2^	Ruminant-associated cases *vs*. controls^3^	Environmental water-associated cases *v*s. controls^4^
Season			
Winter (Dec-Feb)	Reference	Reference	Reference
Spring (Mar-May)	3.78 (1.35–10.59)	0.66 (0.29–1.53)	0.87 (0.23–3.36)
Summer (Jun-Aug)	5.81 (2.23–15.11)	2.94 (1.39–6.23)	1.87 (0.52–6.73)
Autumn (Sep-Nov)	2.96 (1.00–8.73)	1.65 (0.77–3.57)	1.28 (0.33–4.97)
Age group			ns
≤4 years	Reference	Reference	
5–14 years	0.78 (0.36–1.70)	0.62 (0.27–1.45)	
15–24 years	2.52 (1.13–5.59)	0.69 (0.24–2.01)	
25–44 years	2.30 (1.17–4.49)	0.55 (0.23–1.30)	
45–64 years	1.59 (0.79–3.20)	0.40 (0.16–1.01)	
≥65 years	1.13 (0.48–2.68)	0.38 (0.12–1.16)	
Gender (female *vs*. male)	1.11 (0.75–1.64)	0.75 (0.43–1-31)	ns
Urbanization degree			ns
Urban (>1000 people/km^2^)	Reference	Reference	
Intermediate (200–1000 people/km^2^)	0.86 (0.55–1.37)	1.68 (0.79–3.55)	
Rural (<200 people/km^2^)	1.15 (0.71–1.85)	2.09 (1.06–4.13)	
Eating chicken (4.2%)		ns	ns
Summer	Reference		
Autumn	1.69 (0.77–3.70)		
Winter	3.13 (1.07–9.13)		
Spring	1.32 (0.59–2.93)		
Eating poultry other than chicken (8.6%)		ns	ns
Summer	Reference		
Spring	0.56 (0.22–1.38)		
Autumn	2.70 (1.25–5.86)		
Winter	3.28 (1.20–9.01)		
Eating grilled sausages (7.9%)	ns	0.49 (0.27–0.92)	ns
Drinking tap water (7.2%)	0.50 (0.32–0.80)	ns	ns
Contact with fresh produce (5.5%)	0.37 (0.22–0.62)	0.45 (0.23–0.87)	ns
Contact with garden soil (5.4%)	3.18 (2.02–5.02)	2.98 (1.48–5.97)	3.27 (1.33–8.02)
Outdoor sport/recreational activity (4.1%)	0.63 (0.40–0.99)	0.49 (0.26–0.91)	ns
Tap water provider (0.0%)	ns		ns
Local only		Reference	
Local and regional		3.80 (1.22–11.86)	
Regional only		1.36 (0.43–4.34)	

ns = factor not significant (p > 0.05) and/or not influencing the associations of the other covariates, thereby dropped from the multivariable model.

^1^Fraction of imputed missing values in the whole data set.

^2^Based on 161 cases with mean *Pr*_P_ of 0.93 (range 0.83–0.99), and 624 controls.

^3^Based on 68 cases with mean *Pr*_R_ of 0.84 (range 0.81–0.94), and 624 controls.

^4^Based on 21 cases with mean *Pr*_W_ of 0.38 (range 0.28–0.94), and 624 controls.
