# Supplementary material for: Human Campylobacteriosis in Luxembourg, 2010–2013: A Case-Control Study Combined with Multilocus Sequence Typing for Source Attribution and Risk Factor Analysis
Source: Sci Rep. 2016 Feb 10;6:20939. doi: 10.1038/srep20939 (PMC4748240; doi:10.1038/srep20939)
Supplement: Supplementary Information [file srep20939-s1.pdf]

SUPPLEMENTARY INFORMATION S1 AND S3

Human Campylobacteriosis in Luxembourg, 2010-2013: A Case-Control Study Combined with Multilocus Sequence Typing for Source Attribution and Risk Factor Analysis

Joël Mossong, Lapo Mughini-Gras, Christian Penny, Anthony Devaux, Christophe Olinger, Serge Losch, Henry-Michel Cauchie, Wilfrid van Pelt, and Catherine Ragimbeau

Supplementary Information S1

Questionnaire used in the case-control study

1. Informed consent

We thank you that you participate in our study. We would like to draw your attention on certain important aspects.

- Your participation is voluntary.
- The information in this questionnaire will be treated confidentiality.
- This questionnaire contains an anonymous identifier unknown to persons outside of the project that allows us to link your answers with microbiological results of the bacterial isolate in our laboratory
- You can quit the study at any time, even after having sent the questionnaire by contacting us at the address below.
- One of the aims of the study is to publish scientific articles and reports which do not contain identifiable personal information
- All information will be stored for a duration of ten years and the Laboratoire National de Santé certifies that data protection will be guaranteed according to the modified law from 2nd August 2002.

Before you continue with questionnaire, please indicate your consent.

○ Yes, I have read and understood the invitation letter and the informed consent and I agree to participate

○ Yes, I have read and understood the invitation letter and the informed consent and I agree to participate on behalf of my child

○ No, I do not want to participate

Name : ________________________________ Date: _____/____/20____

Signature: ________________________________

2. Data on the person affected by gastro-enteritis

Q2. Today's date? Date: ____/____/20____

Q3. What is the age of the ill person? _________________

Q4. What is the sex of the ill person? ○ Male ○ Female

Q5. Postal code? ___________________

Q6. Does the ill person live in a rural or urban area?

○ Urban ○ Rural ○ A bit of both ○ Don't know

3. Information on illness

Q7. Did the ill person have any of the following symptoms?

	Yes	No	Don't know
Malaise	◯	◯	◯
Abdominal cramps	◯	◯	◯
Diarrhoea (2x or more per 24h)	◯	◯	◯
Fever > 38,5°C	◯	◯	◯
Headache	◯	◯	◯
Bloody stool	◯	◯	◯
Vomiting	◯	◯	◯

Other symptoms or complications: ____

Q8. Start of symptoms: Date : ____/____/20____

Q9. Is the person still ill? ◯ Yes ◯ No ◯ don't know

If no, please indicate duration of illness in days: ___________ days

Q10. Was the person hospitalised? ◯ Yes ◯ No ◯ don't know

If Yes, please indicate duration of hospital stay: ___________ days

4. Recent travel (within 5 days before illness)

Q11. Recent travel abroad? ◯ Yes ◯ No

If Yes, date of return: ____/____/20____

Q12. Destination country?

Country 1: ______________________________________

Country 2: ______________________________________

Country 3: ______________________________________

5. Contacts and activities

Q13. Contacts with domestic animals (within 5 days before illness)?

	No	At home	Outside of home	Don't know
Dogs	☐	☐	☐	☐
Cats	☐	☐	☐	☐
Cows	☐	☐	☐	☐
Pigs	☐	☐	☐	☐
Chicken	☐	☐	☐	☐
Other animal	☐	☐	☐	☐

If other animal, which one: ____

6. Recent activities

Q14. Recent activities (within 5 days before illness)?

	Yes	No	Don't know
Fishing	◯	◯	◯
Swimming	◯	◯	◯
Other water sports	◯	◯	◯
Gardening	◯	◯	◯
Outdoor sports	◯	◯	◯
Recreational activities	◯	◯	◯

If Yes to outdoor activities, please indicate location(s): ____

Q15. Is there an agricultural farm close to the ill person's home?

◯ Yes ◯ No ◯ Don't know

If yes, please indicate approximate distance in metres: _____________ metres

Q16. Are there any pasture for cattle or farm animals close to the home of the ill person?

◯ Yes ◯ No ◯ Don't know

If Yes, please indicate approximate distance in metres : _____________ metres

Q17. Were there recently any flies at the home of the ill person?

◯ No ◯ Yes, a bit ◯ Yes, quite a few ◯ Yes, many ◯ Don't know

Q18. Within 5 days before illness, did the ill person touch with their hands…

	Yes	No	Don't know
Garden soil	◯	◯	◯
Sand (e.g. sand box)	◯	◯	◯
Raw meat	◯	◯	◯
Raw vegetables	◯	◯	◯
Dirty dishes	◯	◯	◯
Recuperated rain water	◯	◯	◯

7. Food and drinks

Q19. Drinking water consumption (within 5 days before illness):

	Yes	No	Don't know
Communal tap water	◯	◯	◯
Bottled water	◯	◯	◯
Water from well or private source	◯	◯	◯

Q20. Milk product consumption (within 5 days before illness):

	No	At home	Outside of home	Don't know
Raw milk from farm	☐	☐	☐	☐
Fresh pasteurized milk	☐	☐	☐	☐
Long-life milk	☐	☐	☐	☐
Cheese made from raw milk	☐	☐	☐	☐

Q21. Meat consumption (within 5 days before illness):

	No	At home	Outside of home	Don't know
Chicken with skin	☐	☐	☐	☐
Chicken without skin	☐	☐	☐	☐
Turkey, duck or other poultry	☐	☐	☐	☐
Beef (steak, entrecote, etc...)	☐	☐	☐	☐
Offal (liver, heart, sweetbreads, tongue, etc...)	☐	☐	☐	☐
Hamburger	☐	☐	☐	☐
Raw minced meat	☐	☐	☐	☐
Cured ham	☐	☐	☐	☐
Cooked ham	☐	☐	☐	☐
Smoked cold sausage (salami,)	☐	☐	☐	☐
Grilled sausages	☐	☐	☐	☐
Liver pâté	☐	☐	☐	☐
Kebab	☐	☐	☐	☐
Lamb/mutton	☐	☐	☐	☐
Meat for grill/barbecue	☐	☐	☐	☐

Q22. Fruit and vegetable consumption (within 5 days before illness) :

	No	Yes	Don't know
Vegetables from own garden	☐	☐	☐
Organic vegetables	☐	☐	☐
Vegetables from shop	☐	☐	☐
Organic fruit	☐	☐	☐
Fruit from shop	☐	☐	☐

We thank you very much for your participation!

Supplementary Information S3

Posterior assignment probabilities of human sequence types to chicken, ruminants, swine, and environmental water. Both the total number of human cases included in the source attribution analysis and those acquired in Luxembourg included in the case-control study are shown.

ST	Total number of cases	Cases included in the case-control study	Posteriori assignment source probability				Assigned source of cases in the case-control study[1]
			Poultry	Ruminants	Swine	Environmental water	
5	3	0	0.955201	0.0395766	0.0000967	0.0051254	
19	50	16	0.153051	0.841061	0.0000259	0.0058621	R
21	91	34	0.18501	0.810502	0.0000272	0.0044614	R
22	5	0	0.231858	0.750783	0.0000357	0.0173237	
25	4	0	0.455497	0.233683	0.0001642	0.310655	
38	1	0	0.0681358	0.924302	0.0000219	0.0075398	
42	20	4	0.185039	0.813091	0.0000264	0.0018433	R
44	28	10	0.920894	0.0632051	0.0000742	0.0158266	P
45	29	0	0.56494	0.39384	0.0000639	0.0411559	
46	8	0	0.206998	0.782649	0.0000259	0.0103271	
47	3	0	0.110191	0.845722	0.0000329	0.0440541	
48	78	0	0.293456	0.700425	0.0000342	0.0060847	
49	5	1	0.962573	0.0109321	0.0001881	0.0263068	P
50	64	0	0.468007	0.515992	0.0000483	0.0159527	
51	24	7	0.93886	0.0557851	0.0000712	0.0052838	P
52	11	1	0.877133	0.0796402	0.0000833	0.0431438	P
53	4	0	0.936776	0.0578041	0.0000708	0.0053494	
61	11	4	0.133116	0.856401	0.000036	0.0104466	R
82	1	0	0.982807	0.0153678	0.00003	0.0017953	
98	1	0	0.687351	0.250771	0.0001958	0.0616826	
104	1	1	0.182823	0.814567	0.0000195	0.0025904	R
110	1	0	0.386704	0.609047	0.0003199	0.0039297	
122	20	5	0.91511	0.0656441	0.0000754	0.0191708	P
161	1	0	0.512744	0.0084651	0.0001197	0.478671	
233	2	1	0.115771	0.877701	0.0000224	0.0065059	R
257	63	0	0.476059	0.51079	0.0000483	0.0131022	
262	2	1	0.0640992	0.934531	0.0000187	0.0013513	R
267	15	0	0.341844	0.629838	0.0000464	0.0282712	
273	1	0	0.309949	0.0287752	0.0002236	0.661052	
290	2	1	0.0597702	0.938988	0.0000187	0.0012232	R
305	5	1	0.938768	0.0558603	0.0000712	0.0053	P
324	1	0	0.462138	0.236921	0.0001658	0.300775	
336	1	0	0.681676	0.312259	0.0000488	0.006016	
350	1	1	0.947195	0.0012649	0.0002002	0.0513404	P
353	2	0	0.996658	0.0015669	0.0000472	0.0017277	
354	39	10	0.905878	0.068447	0.0000776	0.0255976	P
356	18	3	0.462728	0.236633	0.0001656	0.300473	W

367	1	0	0.529464	0.462295	0.0000355	0.0082051	
383	2	0	0.873113	0.0817943	0.0000834	0.045009	
400	9	1	0.938677	0.0559373	0.0000717	0.0053145	P
418	1	0	0.356139	0.614796	0.0000464	0.0290193	
429	2	0	0.983679	0.0042856	0.0000326	0.0120028	
436	2	0	0.931938	0.0010613	0.0003524	0.066648	
441	5	2	0.975555	0.0085665	0.0000899	0.0157883	P
443	2	1	0.939145	0.0555016	0.0000711	0.0052828	P
447	1	1	0.457731	0.23873	0.0001655	0.303373	W
457	1	1	0.993351	0.0004223	0.0000269	0.0061998	P
462	2	0	0.849182	0.013854	0.0001294	0.136835	
464	30	9	0.924876	0.0611112	0.0000742	0.0139391	P
466	1	0	0.983285	0.0130132	0.0000349	0.0036668	
474	2	0	0.265987	0.72945	0.0000233	0.0045395	
475	8	1	0.310194	0.0618469	0.0001358	0.627824	W
492	2	0	0.264625	0.731446	0.0000231	0.0039058	
508	1	0	0.0246929	0.0000045	0.000103	0.9752	
523	2	1	0.938244	0.0563816	0.0000711	0.0053029	P
534	1	0	0.693638	0.0000201	0.0007252	0.305617	
538	1	0	0.904473	0.0064347	0.0000658	0.0890269	
564	1	0	0.603166	0.188237	0.0001907	0.208406	
572	68	0	0.344093	0.618065	0.0000505	0.0377917	
574	1	0	0.996147	0.0024044	0.0000382	0.0014107	
577	1	0	0.15544	0.840061	0.0000188	0.0044801	
583	4	0	0.462027	0.235583	0.0001603	0.30223	
586	3	2	0.0598918	0.938839	0.0000189	0.0012502	R
587	8	1	0.928344	0.003787	0.0001922	0.0676772	P
607	7	3	0.461795	0.235735	0.0001653	0.302305	W
658	3	1	0.460508	0.238014	0.0001643	0.301314	W
677	3	1	0.458163	0.238753	0.0001675	0.302916	W
696	1	1	0.932131	0.0001944	0.0002197	0.067455	P
764	1	0	0.427822	0.0712349	0.0000961	0.500847	
775	8	0	0.334054	0.663412	0.0000353	0.0024988	
824	3	2	0.916384	0.0643775	0.0000758	0.0191628	P
825	8	0	0.904983	0.064012	0.001604	0.0294005	
827	11	3	0.900343	0.0670944	0.0017661	0.0307968	P
828	2	0	0.855228	0.10306	0.0287965	0.0129152	
829	4	0	0.92196	0.0560065	0.0040639	0.0179699	
832	6	1	0.927088	0.0607626	0.0022579	0.0098912	P
854	5	0	0.433084	0.465967	0.0822963	0.0186529	
855	1	0	0.945281	0.0490915	0.0005551	0.0050727	
859	2	1	0.991131	0.0053121	0.0000406	0.0035164	P
860	7	2	0.943328	0.0512915	0.000233	0.005148	P
861	4	0	0.229223	0.651546	0.0001624	0.119069	
870	1	0	0.962046	0.0112492	0.0001865	0.0265181	
872	9	5	0.922264	0.0593414	0.0010153	0.0173791	P
877	1	0	0.909818	0.079978	0.0000819	0.0101216	
879	5	1	0.938516	0.0560513	0.0000709	0.0053616	P
881	2	1	0.975367	0.0013763	0.0006592	0.0225978	P

883	16	4	0.937825	0.0568306	0.0000708	0.0052736	P
892	1	0	0.972899	0.0073952	0.0091964	0.0105096	
904	14	4	0.938764	0.0558058	0.0000712	0.0053586	P
905	1	0	0.938261	0.0563855	0.0000713	0.0052826	
917	2	0	0.468335	0.52621	0.0000364	0.0054185	
918	1	0	0.366538	0.229252	0.0000758	0.404134	
931	2	2	0.991105	0.005322	0.0000406	0.0035326	P
945	1	1	0.71484	0.0013119	0.0012707	0.282577	W
977	4	1	0.938432	0.0561861	0.0000716	0.0053098	P
982	1	0	0.400284	0.59146	0.0000315	0.0082242	
985	1	0	0.32284	0.0394758	0.0905287	0.547155	
989	2	0	0.972474	0.0001461	0.0003456	0.0270347	
990	22	9	0.938766	0.0558535	0.0000713	0.005309	P
991	1	0	0.962387	0.0109944	0.0001896	0.0264286	
1003	4	2	0.960405	0.0003506	0.0000307	0.0392141	P
1033	1	0	0.9624	0.0109498	0.0001883	0.0264618	
1044	10	5	0.704283	0.0142919	0.0001194	0.281306	W
1045	1	1	0.900187	0.0121965	0.0001852	0.0874309	P
1055	1	0	0.913735	0.0712021	0.0087174	0.0063459	
1073	9	2	0.938726	0.0558799	0.0000713	0.005323	P
1096	2	0	0.773457	0.167753	0.0475835	0.0112058	
1103	1	1	0.959744	0.0095022	0.0054328	0.0253209	P
1107	1	1	0.0120695	0.0026528	0.982919	0.0023585	S
1145	1	0	0.45848	0.36572	0.149556	0.0262443	
1181	1	0	0.879599	0.0028844	0.0015917	0.115925	
1191	1	0	0.960023	0.0029022	0.0342096	0.0028647	
1326	2	0	0.274448	0.555112	0.0000987	0.170341	
1359	1	0	0.629174	0.361911	0.0000516	0.0088636	
1374	1	0	0.983436	0.0009434	0.0002895	0.0153312	
1395	1	0	0.229119	0.0474257	0.0001328	0.723323	
1409	3	0	0.965714	0.0229782	0.0000505	0.0112573	
1460	3	1	0.980816	0.0062503	0.0000431	0.0128901	P
1474	1	0	0.478378	0.268418	0.0001096	0.253095	
1519	2	0	0.952102	0.0358317	0.000047	0.0120191	
1579	2	1	0.289751	0.34288	0.34468	0.0226884	S
1582	1	1	0.943656	0.0511426	0.0001463	0.005055	P
1595	5	3	0.916232	0.0605597	0.0030097	0.0201989	P
1614	1	0	0.942287	0.0515788	0.0004531	0.0056816	
1628	3	0	0.580505	0.407661	0.0001578	0.0116761	
1707	1	0	0.93903	0.0555456	0.0000709	0.005354	
1709	4	1	0.93845	0.0561722	0.0000716	0.0053065	P
1721	1	0	0.880159	0.0000639	0.0003097	0.119467	
1728	1	1	0.898808	0.0818198	0.0000478	0.019324	P
1750	4	1	0.943245	0.0513164	0.0002124	0.0052261	P
1759	1	0	0.938739	0.0558851	0.0000713	0.0053047	
1767	2	0	0.974	0.0043111	0.0140968	0.007592	
1801	1	0	0.923137	0.0734177	0.0000512	0.0033945	
1818	1	0	0.562989	0.4034	0.0000439	0.0335663	
1898	1	0	0.991604	0.00595	0.000059	0.0023872	

1919	1	0	0.928777	0.0679915	0.0001054	0.0031259	
1929	1	1	0.996603	0.0023546	0.0000379	0.0010048	P
1943	1	0	0.556594	0.381841	0.0002047	0.0613598	
1947	2	1	0.875395	0.0806585	0.0000836	0.0438628	P
1962	3	1	0.459618	0.239283	0.0001669	0.300931	W
2004	1	0	0.949021	0.0005899	0.0221423	0.0282466	
2036	1	0	0.987447	0.0002134	0.0000641	0.0122753	
2037	2	0	0.998901	0.0007376	0.0000241	0.0003369	
2057	1	0	0.704047	0.287104	0.0000473	0.008802	
2065	3	0	0.992299	0.0066689	0.0000307	0.0010015	
2066	1	1	0.938885	0.0557448	0.0000712	0.0052986	P
2076	1	0	0.773183	0.000066	0.0001531	0.226598	
2122	1	0	0.922798	0.0002683	0.0000686	0.0768653	
2123	1	1	0.960066	0.0267923	0.0000771	0.0130648	P
2133	1	0	0.539358	0.0001705	0.0002524	0.460219	
2135	4	2	0.938186	0.0564672	0.0000709	0.0052762	P
2155	1	0	0.899907	0.0966316	0.0000555	0.0034063	
2167	1	0	0.704236	0.0219232	0.0006311	0.27321	
2176	1	0	0.742584	0.143206	0.0001882	0.114021	
2183	2	0	0.943804	0.05092	0.0001822	0.0050943	
2187	5	2	0.0548992	0.0000334	0.0000546	0.945013	W
2247	1	0	0.288975	0.000113	0.0018291	0.709083	
2258	3	0	0.559397	0.43702	0.0000486	0.0035339	
2274	38	12	0.938742	0.0558938	0.0000712	0.0052928	P
2275	3	1	0.897413	0.0831235	0.000046	0.0194172	P
2288	2	0	0.911249	0.0149528	0.0001285	0.0736695	
2324	3	1	0.986568	0.0113077	0.0000402	0.0020844	P
2331	1	0	0.999585	0.0001365	0.0000248	0.0002539	
2361	1	0	0.962759	0.0108597	0.0001879	0.026193	
2416	1	0	0.596404	0.377822	0.0000453	0.025728	
2438	2	0	0.927124	0.0192838	0.0000531	0.0535394	
2574	1	0	0.938241	0.0563883	0.0000715	0.0052997	
2663	1	0	0.53075	0.442868	0.0000422	0.0263398	
2699	1	0	0.110456	0.0247426	0.85792	0.0068816	
2803	1	1	0.997695	0.0002656	0.0000539	0.0019855	P
2807	1	1	0.938764	0.0558655	0.0000713	0.0052996	P
2813	2	0	0.955095	0.0344265	0.0000471	0.0104315	
2844	1	1	0.998061	0.0006471	0.0000241	0.0012679	P
2861	1	0	0.400773	0.0001653	0.0005072	0.598555	
2882	3	1	0.938792	0.0558258	0.0000716	0.0053103	P
2899	2	0	0.999843	0.0000822	0.0000209	0.0000538	
3015	2	0	0.984911	0.0003894	0.000206	0.0144938	
3016	3	2	0.942943	0.0517225	0.0001967	0.0051373	P
3017	2	0	0.941539	0.0530509	0.0001894	0.0052208	
3140	1	0	0.984947	0.0050103	0.0010236	0.0090195	
3155	10	3	0.93909	0.0555297	0.000071	0.0053098	P
3294	1	0	0.468346	0.522375	0.0000329	0.0092464	
3336	1	0	0.378219	0.410895	0.128487	0.0823992	

3544	1	0	0.938702	0.0559272	0.0000713	0.0052995	
3546	1	0	0.962659	0.0109014	0.0001882	0.0262518	
3547	1	1	0.997052	0.0023373	0.000027	0.0005838	P
3573	1	0	0.877604	0.0147842	0.0001399	0.107472	
3574	13	3	0.952913	0.0326974	0.0000475	0.0143425	P
3633	1	1	0.272725	0.336038	0.0001773	0.39106	W
3720	11	6	0.938465	0.0561558	0.0000713	0.0053079	P
3753	1	0	0.945984	0.0098736	0.0116437	0.0324984	
3769	1	0	0.937839	0.0568023	0.0000708	0.0052883	
3794	1	0	0.74256	0.143294	0.0001881	0.113958	
3985	1	1	0.995576	0.0024116	0.0006811	0.0013317	P
3990	1	0	0.942585	0.0519024	0.0002955	0.005217	
4056	3	0	0.56482	0.142768	0.0001114	0.292301	
4234	1	0	0.556574	0.381809	0.0002047	0.061412	
4279	1	0	0.0020487	0.0000207	0.00000927	0.997921	
4430	2	2	0.938106	0.0564928	0.0000718	0.0053295	P
4684	1	0	0.879499	0.0030665	0.0007164	0.116718	
4685	1	0	0.985204	0.0034027	0.0000668	0.0113261	
4709	5	1	0.508223	0.212105	0.0006148	0.279058	W
4738	1	0	0.237218	0.745162	0.0000283	0.0175913	
4774	1	0	0.996585	0.0023685	0.000038	0.0010082	
4799	1	0	0.9879	0.002341	0.0000345	0.009725	
4806	2	0	0.988168	0.0033075	0.0000448	0.0084793	
4811	1	0	0.973383	0.0000387	0.0000656	0.0265131	
4907	1	0	0.923225	0.0000209	0.0000618	0.0766919	
4949	1	0	0.196582	0.0254047	0.767295	0.0107181	
4950	1	0	0.936967	0.0524761	0.0047583	0.0057984	
4956	1	0	0.941435	0.0528652	0.0004883	0.0052117	
4988	1	1	0.989103	0.0004663	0.0087416	0.0016888	P
5018	10	4	0.058987	0.939784	0.0000186	0.00121	R
5019	1	0	0.999405	0.0001411	0.0000252	0.0004291	
5103	1	0	0.542029	0.284777	0.0001967	0.172997	
5113	1	1	0.960933	0.009825	0.0010447	0.0281969	P
5123	1	0	0.995294	0.0022999	0.0002581	0.002148	
5150	4	2	0.833221	0.094776	0.0002	0.0718027	P
5163	3	0	0.94201	0.052715	0.000129	0.0051462	
5173	1	0	0.364436	0.630956	0.0000231	0.0045842	
5187	2	0	0.948502	0.0461625	0.0004013	0.0049339	
5197	1	0	0.702158	0.0119158	0.0000823	0.285844	
5199	1	0	0.24734	0.0000754	0.0003229	0.752262	
5211	1	0	0.99958	0.0001359	0.0000248	0.0002596	
5272	1	0	0.997913	0.0000198	0.0000468	0.0020206	
5287	1	1	0.994609	0.0002469	0.0009358	0.0042085	P
5288	1	0	0.377758	0.432234	0.0001091	0.189899	
5289	1	0	0.188852	0.799249	0.000028	0.0118707	
5303	2	1	0.900425	0.010532	0.0070529	0.0819898	P
5308	1	0	0.989184	0.002912	0.00009	0.0078138	
5310	1	0	0.900458	0.010521	0.0071297	0.0818917	

5359	1	0	0.670539	0.265308	0.0001967	0.0639572	
5380	3	1	0.926455	0.0566258	0.0016203	0.0152984	P
5493	1	0	0.74256	0.143294	0.0001881	0.113958	
5496	1	1	0.985018	0.0028131	0.0086859	0.003483	P
5498	1	0	0.999711	0.00000211	0.0000402	0.0002468	
5500	1	1	0.879077	0.0030787	0.0007274	0.117117	P
5502	1	0	0.929101	0.0001553	0.000329	0.0704151	
5503	1	0	0.698889	0.0821759	0.0001848	0.218751	
5504	1	0	0.999579	0.0001362	0.0000248	0.00026	
5505	1	0	0.467999	0.230569	0.0004511	0.300981	
5590	1	0	0.589964	0.201203	0.0078193	0.201014	
5633	1	0	0.685349	0.0001281	0.242116	0.0724069	
5636	1	1	0.986507	0.0033808	0.004521	0.0055908	P
5641	1	0	0.962631	0.0108702	0.0001878	0.0263114	
5642	1	0	0.883705	0.074073	0.0002288	0.0419931	
5659	1	0	0.939841	0.0078288	0.0031714	0.0491585	
5746	1	0	0.624905	0.0121516	0.226729	0.136215	
5758	1	0	0.97788	0.0043487	0.0001039	0.017668	
5777	1	0	0.986865	0.0024133	0.0029504	0.0077716	
5778	2	0	0.417479	0.006954	0.0973251	0.478241	
5779	1	1	0.830382	0.13549	0.0000588	0.0340692	P
5780	1	0	0.30408	0.0000236	0.0007854	0.695111	
5782	1	0	0.88079	0.0162949	0.0363486	0.0665669	
5805	1	0	0.461008	0.236028	0.000165	0.302799	
5848	1	1	0.82829	0.0993686	0.0001887	0.0721529	P
5849	1	0	0.967626	0.027995	0.0000448	0.004334	
5850	1	1	0.939315	0.0575915	0.0000485	0.0030452	P
5851	1	1	0.874749	0.117224	0.0000578	0.0079684	P
5852	1	0	0.394636	0.603356	0.0003134	0.0016945	
5853	1	0	0.961883	0.0113876	0.0001856	0.0265433	
5854	1	0	0.894894	0.016317	0.0001129	0.088676	
5855	1	0	0.687351	0.250771	0.0001958	0.0616826	
5959	1	1	0.0944307	0.002638	0.88189	0.0210415	S
5968	1	0	0.996608	0.0023514	0.0000379	0.0010023	
5969	1	0	0.994515	0.0004694	0.0000907	0.0049254	
5972	1	0	0.988955	0.0059149	0.0000325	0.0050977	
5973	1	0	0.962639	0.010906	0.0001887	0.0262658	
5988	1	0	0.458052	0.238654	0.0001665	0.303127	
6131	1	0	0.94369	0.0507726	0.0004432	0.005094	
6173	1	0	0.942395	0.0514936	0.0005637	0.0055481	
6174	1	0	0.881065	0.0028963	0.0013686	0.11467	
6175	1	0	0.877429	0.076125	0.0001548	0.0462916	
6176	1	0	0.895735	0.0004124	0.0018909	0.101961	
6177	1	0	0.996685	0.0000954	0.0001401	0.0030793	
6178	1	0	0.996591	0.002364	0.000038	0.0010072	
6179	1	0	0.760604	0.143737	0.0001915	0.0954673	
6180	1	1	0.998689	0.0002315	0.0000583	0.0010208	P
6279	1	0	0.99791	0.00037	0.0001	0.00162	

6291	1	0	0.381435	0.0136799	0.0002079	0.604677	
6298	1	1	0.981091	0.0089368	0.0009742	0.0089983	P
6302	1	0	0.981505	0.00000905	0.0001836	0.0183029	
